# Impact of PET - CT motion correction in minimizing the gross tumor volume in non-small cell lung cancer

**Published:** 2013

**Authors:** Michael A Masoomi, Anne H McLean, Yassine Bouchareb, Will Ryder, Andy Robinson

**Affiliations:** 1 Department of Nuclear Medicine, Farwaniya Hospital, Kuwait; 2 Nuclear Medicine Physics, Queen Alexandra Hospital, Portsmouth, UK; 3 Clinical Physics, Barts Health - NHS Trust, UK; 4 Faculty of Health Science, University of Sydney, Sydney, Australia; 5 The Harley Street Clinic, London, UK

**Keywords:** Correction, PET-CT, Lung cancer, NCAT

## Abstract

**Objective(s):**

To investigate the impact of respiratory motion on localization, and quantification of lung lesions for the Gross Tumor Volume utilizing a fully automated Auto3Dreg program and dynamic NURBS-based cardiac-torso digitized phantom (NCAT).

**Methods:**

Respiratory motion may result in more than 30% underestimation of the SUV values of lung, liver and kidney tumor lesions. The motion correction technique adopted in this study was an image-based motion correction approach using, a voxel-intensity-based and a multi-resolution multi-optimization (MRMO) algorithm. The NCAT phantom was used to generate CT attenuation maps and activity distribution volumes for the lung regions. All the generated frames were co-registered to a reference frame using a time efficient scheme. Quantitative assessment including Region of Interest (ROI), image fidelity and image correlation techniques, as well as semi-quantitative line profile analysis and qualitatively overlaying non-motion and motion corrected image frames were performed.

**Results:**

The largest motion was observed in the Z-direction. The greatest translation was for the frame 3, end inspiration, and the smallest for the frame 5 which was closet frame to the reference frame at 67% expiration. Visual assessment of the lesion sizes, 20-60mm at 3 different locations, apex, mid and base of lung showed noticeable improvement for all the foci and their locations. The maximum improvements for the image fidelity were from 0.395 to 0.930 within the lesion volume of interest. The greatest improvement in activity concentration underestimation was 7.7% below the true activity for the 20 mm lesion in comparison to 34.4% below, prior to correction. The discrepancies in activity underestimation were reduced with increasing the lesion sizes. Overlaying activity distribution on the attenuation map showed improved localization of the PET metabolic information to the anatomical CT images.

**Conclusion:**

The respiratory motion correction for the lung lesions has led to an improvement in the lesion size, localization and activity quantification with a potential application in reducing the size of the PET GTV for radiotherapy treatment planning applications and hence improving the accuracy of the regime in treatment of lung cancer.

## Introduction

Lung cancer is the leading cause of tumor-related deaths (1). Although rates of bronchial carcinoma–related death in men have decreased on average by 1.8% annually during the past decade, the incidence of lung cancer in women is increasing (1). Non–small cell lung cancer (NSCLC) accounts for about 80% of bronchogenic malignancies. Positron emission tomography (PET) is highly sensitive for tumor tissue in NSCLC studies ^18^F-fluorodeoxyglucose (FDG) PET allows tumor metabolism to be visualized and so can be used together with CT imagesin radiotherapy treatment planning to integrate anatomic and metabolic information which will influence the definition of the gross tumor volume (GTV).

One of the main challenges affecting quantification in PET-CT imaging is the uncontrollable motion of the organs, which results in an inaccurate quantification of the uptake of the tracer in the tumor lesions. Respiratory motion may result in more than 30% underestimation of the SUV values of lung, liver and kidney tumor lesions (2, 3). Motion induced-artifacts on PET reconstructed images caused by the discrepancy between breathing protocols of PET and CT scans can also significantly affect the quality of the attenuation corrected PET images. Respiratory motion affects all tumor sites in the thorax and abdomen (even the pelvis) (4-6), though the disease of most prevalence and relevance for radiotherapy is lung cancer. It is important to note that respiratory motion is just one potential source of error in radiotherapy. Other important errors, particularly for lung tumors, are GTV and clinical target volume (CTV) definition variations and setup errors. Large inter-physician GTV variations for lung cancer (7-9) have been published. Respiratory motion varies from day to day, and tumor and normal tissues can shrink, grow, and shift in response to radiation therapy and potentially other concomitant therapies. The respiration-motion management techniques not only affect the accuracy of target localization, but can also play a role in normal tissue sparing (10, 11). It is also important to note that fast tumor shrinkage occurs quite often in lung radiotherapy, which may give rise to systematic delivery errors (12).

A number of respiratory motion correction techniques have been proposed, however their potentials are not fully evaluated (13, 14). The main limitations of these techniques is that theyare either too slow or suffer from a limited accuracy due to the presence of multiple lesions in different locations within the lungs, resulting in severe artifacts in the image sets. These artifacts originate from patient movements, either voluntary or intrinsic organs motion, adding to the imperfections of the imaging devices. Therefore, unless differences in the scanning protocols and motion issues are considered, image quality and data processing for detecting of abnormalities and assessing the severity of diseases are compromised.

Research studies have shown that respiratory gating during PET imaging reduces the lesion volume and improves the accuracy of quantifying radionuclide uptake. Respiratory gating involves the administration of radiation (during both imaging and treatment delivery) within a particular portion of the patient’s breathing cycle, commonly referred to as the “gate.” Respiratory gating is currently under study by several centers to account for respiratory motion during radio-therapy of thoracic and abdominal tumors (15-22). The study by Boucher *et al* (3) utilizing physical spherical ^18^F-FDG phantoms and simulated respiration, showed an underestimation of the activity concentration for all spheres, with the underestimation increased as the lesion size decreased. Nehmeh *et al* (13)performed respiratory gating for five patient studies and showed the gating consistently reduced the lesion volume measured from the PET scan. The study also showed an increase in the standard uptake value measured within the lesions.

This study describes an investigation into the potential of motion correction for the frames within the respiratory cycles and its effect on the improvement in the reduction of the lesion size and the lesion activity quantification, using the Auto3DReg program that is a voxel-intensity-based and a multi-resolution multi-optimization (MRMO). The NCAT or NURBS (Non Uniform B Splines) based CAT (Cardio Torso Phantom)developed by Segars (23) which simulates both respiratory and cardiac motion was also utilized in this study.

## Methods

The NURBS that is a bidirectional parametric representation of an object was utilized to generate CT attenuation maps and activity distribution volumes as a series of transaxial slices for the lung region. The NURBS surfaces define the respiratory structures in the spline based NCAT, which can be altered via affine/other transformations through manipulation of control points, to simulate respiration. The activity distribution in the organs was based on literature review, evaluating uptake in the various healthy organs (24, 25). Spherical lesions with the various sizes, 20-60 mm, were generated using a separate program within the NCAT phantom and incorporated intothe NCAT phantom in the base of the right and the left lungs. The positions of the lesion were chosen to reflect those areas most likely to be affected by motion. Higher relative activity concentration was assigned to the lesions than to the surrounding lung tissue and organs. The relative concentration was expressed rather than lesion to organ activity ratio to create images that will be quantitatively more descriptive and qualitatively comparable to clinical images (Table 1). To simulate the effect of the point spread function (PSF) of a real PET scanner on the activity images, each image volume was convolved with a Gaussian Kernel as this has been shown to be a good approximation to the true PSF of a PET scanner.

**Table 1:** Relative activity concentrations assigned to NCAT phantom for lung region (24, 25).

Organ	Relative activity Concentration	oran	Relative activity Concentration
Body background	1.00	Liver	1.86
Myocardium	4.44	Gall bladder	1.86
Heart blood pool	1.42	Stomach	1.99
Lung	0.60	Spleen	1.26
Lung lesions	5.60	Spine	2.00

### 

#### Respiratory cycle and frame generation

Five frames were generated throughout the respiratory cycle using the NCAT phantom with normal breathing parameters (Table 2). Therespiratory motion was incorporated by applying affine transformations to the different respiratory structures. The period of the respiratory cycle for normal tidal breathing was 5 seconds with inspiration lasting approximately 2 seconds and expiration lasting the remaining 3 seconds. The normal parameters were based on respiratory mechanics and a respiratory-gated CT data set of a normal patient (23). The extent of inspiration or expiration for each frame is given by the NCAT phantom. The phantom generates matching 3D distribution of attenuation coefficients for a given photon energy (i.e. 511keV) and a 3D distribution of emission radionuclide images corresponding to each of these five frames.

**Table 2:** Respiration parameters used for NCAT phantom generation.

Parameter	Value
Length of respiratory cycle	5 seconds
Time per frame	1 second
Frame 1 start point	Full exhalation
Max diaphragm motion	2 cm
Max anterior-posterior expansion	1.2 cm

#### Respiratory motion correction

**Figure 1:**
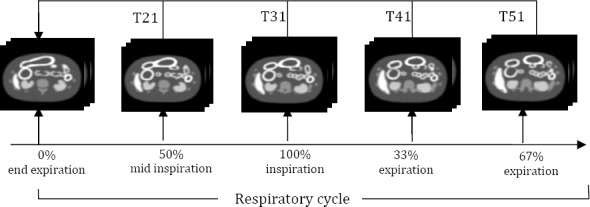
Sketch of the motion correction methodology to estimate the inter-frame 3D motion parameters (T21, T31, T41 and T51); T21 is the transformation which aligns frame 2 (50% inspiration) to the end-expiration frame (reference frame); T31 is the transformation describing the 3D motion between frame 3 (full-inspiration) and the reference frame, etc. To speed-up the motion correction process, transformation T21 is used to initialise the estimation of T31 and the same for the remaining frames.

The motion correction technique used in this study (Figure 1) is an image-based motion correction approach; it uses of a voxel-intensity-based and a multi-resolution multi-optimization (MRMO) algorithm, first described by Bouchareb *et al* (37). The technique takes advantage from the convergence behavior of non-derivative optimization methods when aligningimage sets realized in extreme differences in term of breathing protocols during data acquisition. The key ideas of non-rigid voxel intensity based registration technique consist of multi-resolution and hierarchical transformation model that uses similarity measures to obtain an optimum solution.

The program reads in a CT transmission volume (reference), followed by a second CT trans-mission volume (floating) and an emission volume that was acquired at the same respiratory point as the floating volume. The floating transmission and emission volumes are registered to the reference volume and the motionparameters are estimated. The hierar-chical model first performs rigid body transfor-mations on a low resolution image; named floating image (image to be registered) to align it to a reference image before performing non-rigid transformations to further optimize the alignment. At each hierarchical level (in practice three resolution levels are sufficient) a different image resolution is used, and the original image volumes are resampled (2 mm × 2 mm × 2 mm, 4 mm × 4 mm × 4 mm, and 8 mm × 8 mm × 8 mm are used at the 3 resolution levels), the first level of motion estimation uses the lowest resolution level and subsequent levels move to the finer resolutions. The program implements similarity measures when registering the two image volumes. A sum of squared differences criterion measure was used in this study.

**Figure 2:**
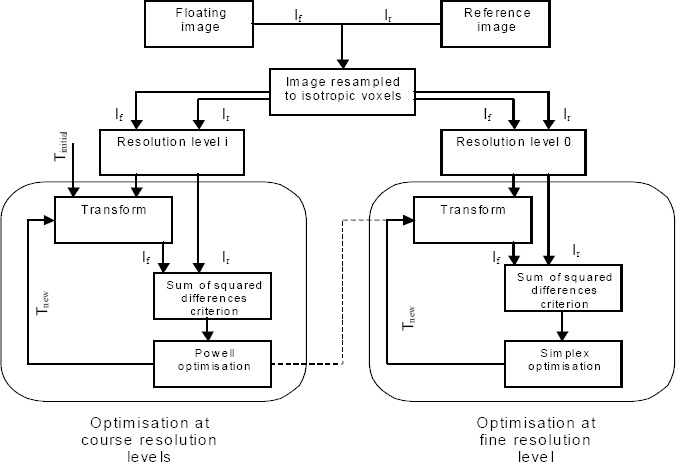
Diagram of Multi-resolution Multi-optimisation algorithm using a hierarchical transformation model and SSD similarity. I_f_ and I_r_ represent floating and reference images, T_initial_ and T_new_ represent transformation parameters.

The MRMO algorithm uses a multi-optimization scheme at each resolution level. The Powell’s method was used at lower resolution levels and the Nelder-Mead simplex optimization was used at the full resolution level (26-27). The simplex method was initialized with the optimal solution found by Powell’s method at the previous resolution. A detailed diagram of the MRMO algorithm is shown in Figure 2. It can be seen that the Powell optimization method is used first at lower image resolutions (left hand side), and when an optimum solution is found this is used to initialize the optimization using a downhill simplex optimization algorithm at the highest resolution level.

All generated frames were co-registered to a reference frame using a time efficient scheme. The reference frame is considered to be the end-expiration frame because it is the most reproducible phase in breathing cycles.

Prior to registration of the frames, each phantom was saved with 3.125 mm voxel size and coded on16-bits Analyze format using the MRIcro image viewing software (28). Frame 1 of the NCAT phantom volume (end-expiration) wastaken to be the reference frame, and the following frames 2 - 5 were individually regist-ered to frame 1.

#### Image Analysis

Following registration of target frames 2 - 5 to the reference frame, the resulting five frames were averaged using the ImageJ (29), an image processing and analysis tool to produce a *motion corrected activity distribution* image. The NCAT phantom generates an average image in addition to the separate frames 1 - 5. This average image represents the activity distribution over thewhole respiratory cycle and it was considered to be the *uncorrected activity distribution* image.

The ImageJ and AMIDE (30) were utilized to compare the uncorrected and corrected activity distribution images through visual assessment, line profile analysis, measurement of lesion dimensions and lesion activity quantification. The activity concentration was measured within the lesions using three different volumes of interest (VOI) definitions. The first spherical VOI was chosen to have half the known diameter of the lesion. Although, the approach is not a feasible VOI definition in practice, this definition was chosen for the purpose of the study to allow the lesions to be compared before and after correction using volumes of the same size. The second and third VOIs were defined using 50% and 80% thresholds based on the maximum voxel value within the lesion. The regions of interest therefore, had different volumes before and after correction. Although SUVmax is often considered as the preferred choice for tumor uptake, but due to presence of noise in any real imaging situation, the SUVmax could be highly variable and usually provides an overestimate of the true maximum pixel value and can also occasionally even underestimate it (32). The threshold values were chosen based on literature reviews (31, 32) and the earlier study by the authors (33) utilizing combination of in-house software analysis and visual assessment. A 3D region growing algorithm based on Boucek *et al* work (34) that will minimize the error is under development and can be applied in future work.

In addition to comparing the average images over the whole respiratory cycle, the improve-ment for individual frames was assessed before and after registration, using image fidelity factor (FF) and correlation factor (CF) defined as follows (35):


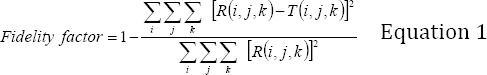



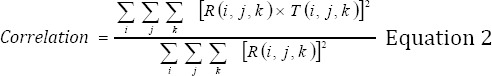


where R(i, j, k) = activity concentration in voxel (i, j, k) of the reference frame and T(i, j, k) = activity concentration in voxel (i, j, k) of the target frame. The image FF is one measure of image quality that is not user dependent. It is derived from the normalized sum of the square difference of voxel values (I, j, k) of a measured image (T) and a reference image (R). The image CF is based on the correlation between the measured image (T) and the reference image (R). The FF and CF take values between 0 and 1(value 1 meaning that the image fidelity and correlation are perfect).

The factors were calculated over two different volumes using an in-house written program. The first volume was chosen to cover the whole image volume, but excluding slices that contained empty voxels after the registration shift. The second was a cuboidalVOI completely surroun-ding the lesion throughout the full extent of itsmotion.

## Results

### 

#### Registration Parameters

For each registration, the software generates the applied transformation parameters to match the target frame to the reference frame.Table 3 shows an example of the parameters for a 20 mm lesion. In general the largest estimated motion is the translation in the z-direction (inferior-superior), followed by the translation in the y-direction (anterior-posterior). The translation is greatest for the frame 3 which is at end-inspiration and smallest for the frame 5, which is closest to the reference frame at 67% expiration.

**Table 3:** The registration parameters for frames 2-5 for a 20 mm diameter lesion in the central base of the right lung.

Parameter	Direction	Target fraine (registered to reference frame 1)			
		Frame 2	Frame 3	Frame 4	Frame 5
Translation	x	0.607	0.554	0.547	0.354
	y	-3.402	-7.660	-6.278	-1.347
	z	8.465	22.887	17.560	1.690
Rotation	x	0.023	0.046	1.455	0.119
	y	-0.017	-0.007	-0.859	0.064
	z	0.083	0.051	-0.027	-0.035
Scaling	x	0.999	0.998	0.999	1.000
	y	0.969	0.932	0.943	0.991
	z	1.049	1.186	1.142	0.992
Shearing	x	-0.183	-0.101	-0.114	0.055
	y	0.071	0.201	0.982	0.123
	z	-0.302	-0.021	1.210	0.344

#### Visual Assessment

**Figure 3:**
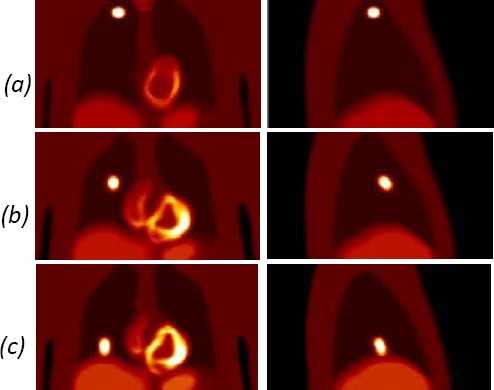
Activity distribution images over whole respiratory cycle showing blurring due to respiration for lesions positioned at (a) apex of lung; (b) mid lung; (c) base of lung.

**Figure 4:**
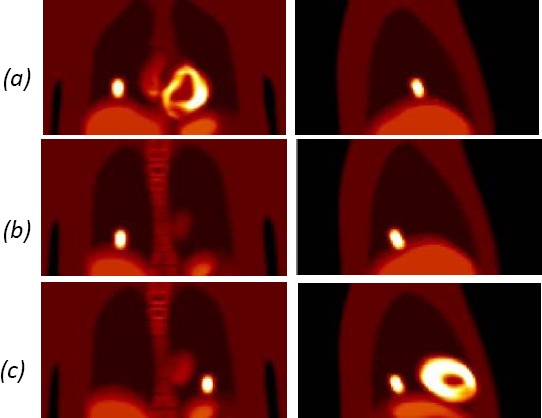
Activity distribution images showing position of 20 mm lesions: (a) central base of right lung; (b) posterior base of right lung; (c) posterior base of left lung.

Initially, the phantoms were generated with lesions in various positions to visually assess the extent of blurring in comparison to a motion free image. Lesions in the range of 20-60 mm diameters were generated in different location in the lung to compare effect of the motion correction (Figure3). The blurring due to respiration was greatest for the lesions in the base of the lung where follow-up assessment of the motion correction was concentrated. As such lesions of 20 mm were generated in the three different locations (Figure 4): central base of the right lung; posterior base of the right lung and posterior base of the left lung. Figure 4 shows the effect of respiratory motion for the 20 mm diameter lesions within the lung.

**Figure 5:**
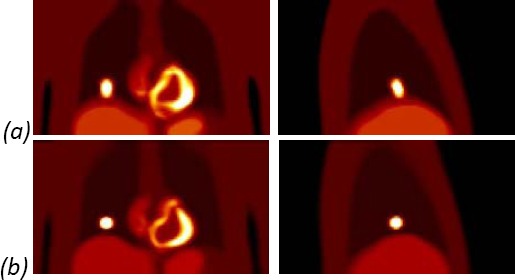
The average activity distribution in all five frames for a 20 mm lesion (a) before registration of frames; (b) after registration of frames.

**Figure 6:**
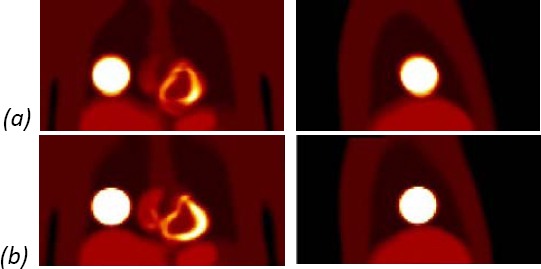
The average activity distribution in all five frames for a 60 mm lesion (a) before registration of frames; (b) after registration of frames.

For each phantom the improvement was initially assessed visually, by comparing the uncorrected activity distribution image and the motion corrected image. The improvement was noticeable for all the lesion sizes and locations (Figures 5-6). In addition to reduction in size, the lesions were more distinct from the liver.

#### Line Profile Analysis

**Figure 7:**
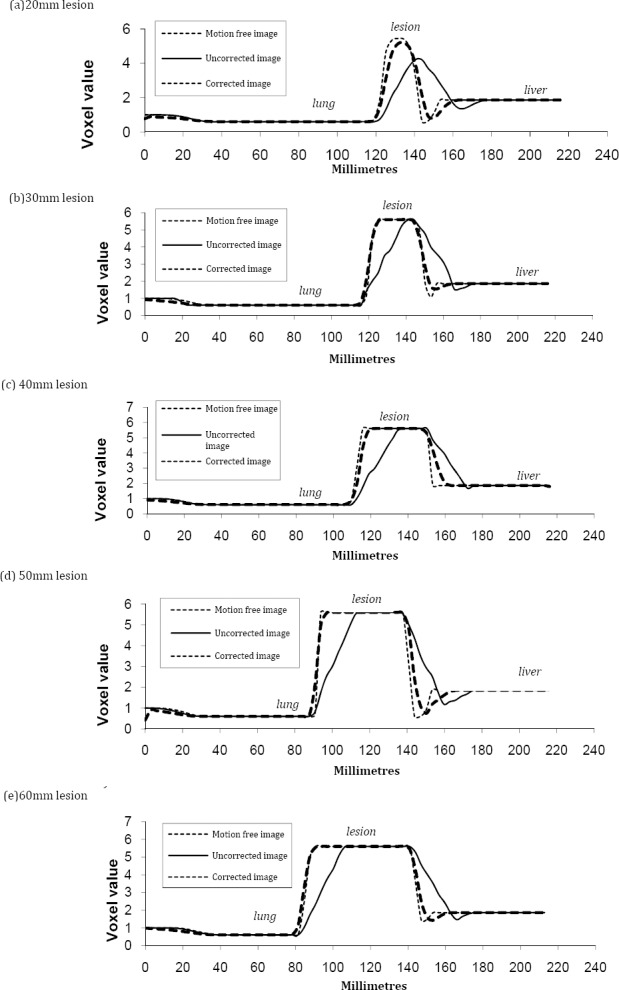
Line profiles in the superior-inferior direction for different lesion diameters in the central base of the right lung.

The improvement of activity within the lesion was assessed using line profiles. Figure 7 shows the superior-inferior profiles for the 20 – 60 mm lesions in the central base of the right lung. Ideally, the profile for the motion corrected image should match the profile for the motion free image generated without respiratory motion.

Improvement, following the motion correction was observed for all the lesion sizes, with the profile being closer to the motion free profile. The profile for the 20 mm lesion shows that maximum activity and the lesion size are improving significantly following motion correction, which is due to reduced blurring of the activity within the lesion. Furthermore, the background activity distribution between the lung and the liver is lower after correction due to better distinction of the two features. For all the other lesion sizes, the maximum activity seen in the corrected and uncorrected profiles is equal to the true maximum activity seen in the motion free profile. However, an improvement is seen in the shape of the lesion peak, which is squarer after correction as lesions are experiencing less blurring at the edges.

#### Measurement of Lesion Dimensions

**Figure 8:**
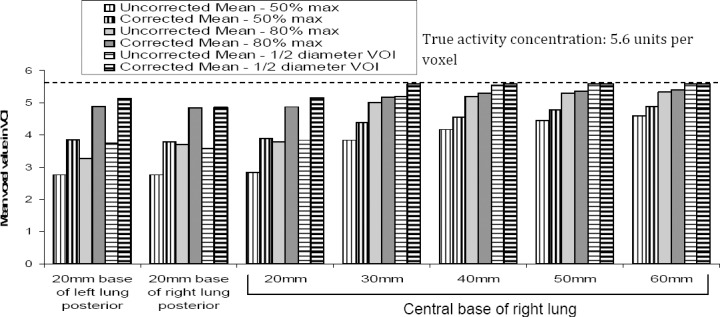
The reduction in lesion dimensions following registration of frames 2-5 to the reference frame. The difference was measured between the average uncorrected and corrected activity distribution images.

The lesion dimensions in the anterior-posterior, inferior-superior and lateral directions were measured, based on FWHM and FWTM, before and after registration of frames using the average activity distribution images. In all cases, the lesion dimensions were reduced following the motion correction (Figure 8). The greatest improvement was achieved in the inferior-superior direction (4.5-13.8 mm) and tends to be smallest in the lateral direction where there is the least motion to respiration. The improvements of 0.9 - 8.1 mm for the anterior-posterior and 0.4 – 2.2 mm for the lateral directions were also achieved.

#### Fidelity and Correlation Factors

**Figure 9:**
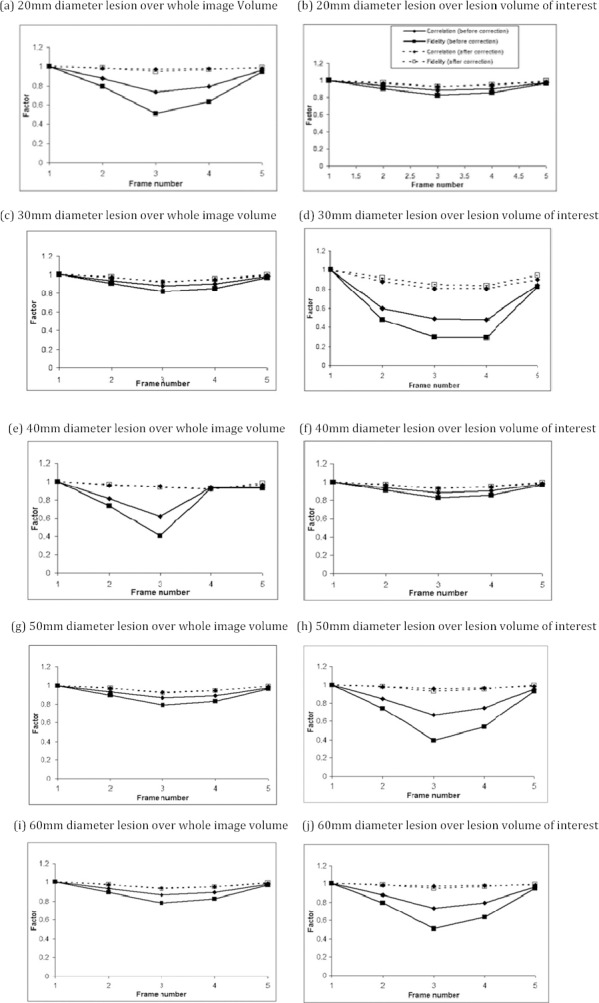
Improvement in image fidelity and correlation factors before and after registration of each frame to the reference frame. The comparison is made between each frames, 1-5, and the reference frame 1.

The extent of the improvement was assessed on a frame-by-frame basis in comparison to the reference frame using the image FF and CF. Figure 9 shows the results for the 20 – 60 mm diameter lesions, indicating improvement in the fidelity and correlation factors for all the lesions. The FF and CF were lowest for the frame 3, which is at full inspiration and therefore furthest from Figure 6. The average activity distribution in all five frames for a 60 mm lesion (a) before registration of frames; (b) after registration of frames.

frame 1. For all the lesion of sizes 10, 20, 30, 40, 50 and 60 mm which were incorporated into the NCAT phantom in the base of the right lung, the improvement is more significant for the localized rectangular volume surrounding the lesion. The maximum improvement in fidelity from 0.395 to 0.930 was calculated for a lesion within the volume of interest. Future work to investigate the localized motion correction is appearing to be informative.

#### Assessment of Lesion Activity Concentration

**Figure 10:**
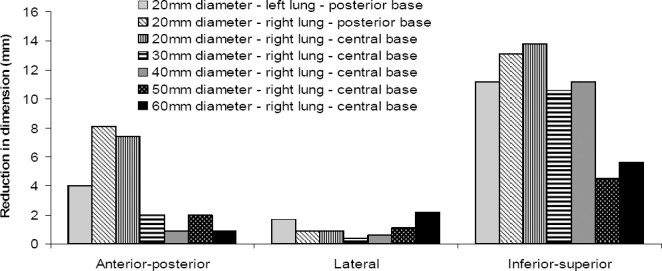
Mean voxel value within each lesion volume of interest for the uncorrected and corrected activity distribution images, using three different volume definitions. The true activity concentration is shown by the dashed line.

For all methods of defining ROI over the lesion sizes, an improvement in activity quantification following motion correction was observed (Fig10). The greatest underestimation of activity concentration was noted down for the 20 mm lesions although, the greatest improvements were observed following registrations of the frames. In general, as the lesion size is increasing, the activity underestimation is decreased whichis in agreement with the study performed by the Boucher *et al* (3).

#### Overlay of Activity Distribution on Attenuation Map

**Figure 11:**
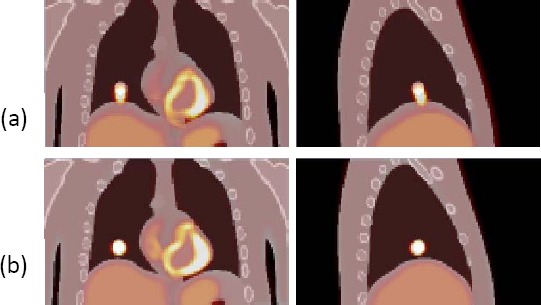
Overlay of activity distribution on reference frame attenuation map for: (a) the uncorrected average activity distribution; (b) the motion corrected average activity distribution.

Following the motion correction of the frames, the lesion in the activity distribution image coincided better with the lesion in the attenuation image (Fig11), indicating that the image registration technique, enables improvement in the localisation of PET metabolic information relative to an anatomical CT image.

## Discussion

Respiratory motion affects all tumor sites in the thorax and abdomen (even the pelvis) (4-6), motion varies from day to day, and tumor and normal tissues can shrink, grow, and shift in response to radiation therapy and potentially other concomitant therapies.

A number of respiratory motion correction techniques have been proposed, however theirpotentials are not fully evaluated (13, 14). The fully automated Auto3Dreg algorithm (a voxel-intensity-based and a multi-resolution multi-optimization) together with the generated digitized images (using NCAT anthropomorphic phantom) were utilized to investigate and assess the impact of respiratory motion on lesions at the base of the lung where they are more subject to motion variation.

The largest motion was observed for translation in z- direction (inferior-superior) followed by translation in anterior-posterior. The translation was greatest at the end inspiration and at 67% expiration.

Mean activity concentration within 20 mm lesion VOI increased with deviation from true value; - 31.4% to -7.7%.

The blurring due to respiration was greatest for 20 cm lesions in the base of the lung. The motion correction of lesions <10 mm has not been addressed and requires further investigation.

The line profile analysis of activity distribution post motion correction exhibited distinct improvement of the lesion activities for all lesions. Improvement in the Lesion dimensions was measured to be the largest in the inferior–superior (4.5-13.8 mm) and the smallest in the lateral direction (0.4 -2.2 mm) where there is least motion.

The extent of motion correction on image quality improvement was further assessed on frame by frame assessment based on FF and CF calculation. Improvements were observed for all the lesions, with the lowest FF and CF at full inspiration, i.e. frame 3. The maximum improvements for FF and CF (0.395 to 0.930) were recorded.

The percentage threshold method was chosen, based on literature review, previous work and the expert assessment. A drawback of his approach is that the resulting region may depend on how much noise is present, as the isocontour value is often based on the maximum pixel values in ROI or tumor and furthermore if contour percentage is too low, the resulting region may spread out to inadvertently include a significant portion of the background (31). To bypass the problem a 3D region-growing algorithm can be used (34).

If the motion due to respiration is known by characterising motion parameters derived from gated images, it would be possible to incorporate these parameters into the reconstruction of the entire PET data set as demonstrated by Lamere *et al*. (36). This would enable respiration to be corrected for as part of the image reconstruction process rather than reconstructing individual gates of data. The main advantage of this is the improved statistics of reconstructing the whole data set with many recorded events, than many gates with fewer events per gate. The effectiveness of the approach is yet to be tested on real (as opposed to anthropomorphic phantom) images.

Further work is required to assess the effectiveness of MRMO technique with extreme breathing parameters. For normal inspiration (used in this work) the maximum diaphragm movement is set to 2 cm and the maximum anterior-posterior expansion is set to 1.2 cm. These parameters could easily be increased and would create data with larger motion artifacts in all frames.

A more realistic method of creating activityphantom images for comparison would be to either convolve the raw NCAT data with a spatially variant point spread function (PSF) for a real scanner; or to simulate the acquisition process using Monte Carlo methods (e.g.SimSET, GATE, etc.) to more realistically simulate PET images. This would allow the motion correction techniques to be assessed for suitability with realistic conditions before assessing clinical data. It would also be beneficial to test this method using a PET-CT respiratory phantom such as the Quality Assurance System for Advanced Radiotherapy (QUASAR) with the optional PET-CT insert.

## Conclusion

The respiratory motion correction for the lung lesions has led to an improvement in the lesion size, localization and activity quantification with a potential application in reducing the size of the PET GTV for radiotherapy treatment planning applications and hence improving the accuracy of the regime in treatment of the lung cancer.
